# Comparison of endoscopic and endoscope-assisted microscopic transsphenoidal surgery for pituitary adenoma resection: a prospective randomized study

**DOI:** 10.3389/fendo.2025.1552526

**Published:** 2025-07-22

**Authors:** Márton Eördögh, László Bárány, Christian Rosenstengel, Victoria Bogaczyk, Jörg Baldauf, Silke Vogelgesang, Andreas Stahl, Michael Kirsch, Werner Hosemann, Antje Steveling, Ahmed Al Menabbawy, Henry W. S. Schroeder

**Affiliations:** ^1^ Department of Neurosurgery, University Medicine Greifswald, Greifswald, Germany; ^2^ Department of Neurosurgery, University Hospital Erlangen, Friedrich-Alexander-University, Erlangen, Germany; ^3^ Institute of Pathology, University Medicine Greifswald, Greifswald, Germany; ^4^ Department of Ophthalmology, University Medicine Greifswald, Greifswald, Germany; ^5^ Institute of Diagnostic Radiology and Neuroradiology, University Medicine Greifswald, Greifswald, Germany; ^6^ Department of Ear, Nose and Throat Diseases, Head and Neck Surgery, University Medicine Greifswald, Greifswald, Germany; ^7^ Department of Internal Medicine “A”, University Medicine Greifswald, Greifswald, Germany

**Keywords:** endonasal, pituitary, pituitary adenoma, transsphenoidal surgery, endoscopic transnasal approach

## Abstract

**Objective:**

The value of endoscopic versus microsurgical approach has not yet been defined in transsphenoidal pituitary adenoma surgery. In this study, we compare both methods and analyze the long-term surgical, radiological, endocrinological, ophthalmological, and rhinological results as well as the patients’ quality of life.

**Methods:**

A total of 33 individuals with elective transsphenoidal pituitary adenoma surgery were randomized (pure endoscopic approach or endoscope-assisted microscopic approach) and prospectively underwent investigations with a focus on patient-related subjective outcome measurements.

**Results:**

The mean follow-up period was 6.3 years. In the microsurgical group, endoscopic inspection revealed residual tumor in seven of 15 patients (46.7%) not seen by the microscope. Endoscopic resection provided long-term tumor-free state in all of them. Compared to pure microsurgical treatment, endoscopy was associated with a lower probability of tumor recurrence (OR = 0.24) and appeared advantageous in the long-term achievement of any surgical goal (OR = 3.80) as well as in anterior pituitary lobe function improvement (OR = 1.60). Where gross total tumor resection was the stated preoperative goal, there was no long-term tumor recurrence in 81.8% (endoscopy group) and 83.3% (endoscope-assisted microsurgical group). Most aspects showed no significant difference between the techniques, such as length of hospital stay, complication rate (endoscopy: 16.7%, endoscope-assisted microsurgery: 20.0%), long-term maintenance of any preoperatively stated extent of resection, pituitary and olfactory function, rates of DI and SIADH, ophthalmological improvement, and SNOT scores.

**Conclusions:**

Both techniques provide good long-term surgical, radiological, endocrinological, rhinological, and ophthalmological results. Endoscopy clearly improved the rate of long-term achievement of the initial surgical goal and the anterior pituitary lobe function.

## Introduction

Decades after Schloffer’s (1) and Hirsch’s (2) first attempts to remove a pituitary tumor, transnasal neurosurgery became inseparable from the microscope (3–5). Nowadays, panoramic endoscopic visualization—introduced by Jankowski et al.—with angled optics excels the microscopic image which is further narrowed by retractors (5–10). Endoscopy seems advantageous concerning not only the extent of tumor resection (8, 11–15) or rhinological outcome (11, 12) but also the length of hospital stay (6, 12, 16). However, the value of endoscopic transsphenoidal pituitary surgery compared to microscopic techniques remains a subject of debate, as the evidence is mostly based on retrospective studies, and prospective, randomized analyses on this topic are rare (8, 17–21). There is also a need for investigations where patients’ subjective measurements (PROM) are confronted with the objective clinical data. To address this, we present our monocentric long-term prospective study of 33 consecutive randomized individuals who underwent endoscopic (E) or endoscope-assisted microscopic (M) transsphenoidal pituitary tumor resection.

## Materials and methods

### Patient population

This study involves patients who underwent elective transnasal pituitary adenoma surgery in our department between 2010 and 2011. A total of 40 adults were assigned to the E or M group by simple randomization. Rathke cleft cysts, active malignancies, emergency or revision surgeries, and extended endonasal approaches were excluded. Within the study period, both techniques were regularly performed by the surgeon (HWSS) in our department. **Table 1** gives a detailed description of the investigated aspects of the follow-up examinations.

**Table 1:** Study agenda.

Schedule	Neurological examination	Imaging^a^	Ophthalmology examination^b^	Rhinology examination^c^	Endocrinology examination^d^	Quality of life^e^	Standardized questionnaire^e^
Prior to surgery	✓	MRI, CT	✓	Examination, endoscopic inspection, rhinomanometry, olfaction test	✓	SF-36, SNOT	✓
3 days	✓	MRI, CT			✓		✓
5 days	✓		✓	Examination, endoscopic inspection			✓
2 weeks	✓			Examination, endoscopic inspection			✓
3 months	✓	MRI	✓	Examination, endoscopic inspection, rhinomanometry, olfaction test	✓	SF-36, SNOT	✓
6 months	✓		✓	Examination, endoscopic inspection	✓	SF-36	✓
1 year	✓	MRI	✓	Examination, endoscopic inspection, rhinomanometry, olfaction test	✓	SF-36, SNOT	✓
2 years	✓	MRI	✓	Examination, endoscopic inspection, olfaction test	✓	SF-36, SNOT	✓
Every 1 to 2 years	✓	MRI	✓	Examination and, as needed, endoscopy, etc.	✓		✓

The evaluation was done by the first author who was not involved in the patient care. Tumor-independent alterations were distinguished. The table describes the timepoints of the given investigations.

a Radiological assessment (performed by neuroradiologists): contrasted MRI and/or CT was performed. GTR (EOR: 100%), NTR (EOR: >95%), and STR (EOR: <95%) were distinguished. The cavernous sinus and suprasellar expansions were described with Knosp and Hardy classification. The tumor size was calculated using the formula: a·b·c·π/6, a–c corresponding to the largest diameter in the main planes.

b Ophthalmological assessment (performed by ophthalmologists) included specific anamnesis, visual acuity tests, and Goldmann visual field tests.

c Rhinological assessment (performed by rhinologists) included specific anamnesis (including earlier rhinological therapies and surgeries), endoscopic inspections, olfaction tests (Sniffin’ Sticks, Burghardt GmbH, Wedel, Germany), and rhinomanometry. An olfaction test score of 10–12 was considered normosmia, 6–9 as hyposmia, and <6 as anosmia.

d Endocrinological assessment (performed by neuroendocrinologists): Prior to surgery, we examined routine serum, urine electrolyte, and hormone levels as well as dynamic hormonal tests. We defined hormonal remission at ACTH-producing tumors as morning serum cortisol of <5 μg/dL (or <138 nmol/L); at TSH-producing tumors, serum TSH/fT4 normalize; at PRL-producing tumors, dilutional serum PRL normalizes; at GH-producing tumors, the random serum GH level is <1.0 μg/L (or <0.4 μg/L in OGTT). Age-adjusted IGF-1 is normalized. Additionally, dynamic tests were conducted. At given time points, the hormonal axes were defined as overfunctioning, underfunctioning, or intact.

e Quality of life: The patients filled out the SF-36 and SNOT-22 questionnaires at given time points. Another form (standardized questionnaire, see **Supplementary Table S1**) was discussed with the patient at each visit.

### Ethics and human rights

All procedures were in accordance with the Helsinki declaration and its amendments. A local ethic committee approval was obtained (BB-38/09). The patients enrolled in this study gave informed consent for study participation.

### Endoscopic binostril transnasal paraseptal transsphenoidal approach

After putting the patient in a supine position, adrenaline-impregnated pledgets were inserted toward the sphenoethmoidal recesses. The 0°-endoscope was introduced. The turbinates were lateralized. The posterior nasal septum was incised, then detached from the sphenoid rostrum, and displaced with the contralateral mucoperiosteum. The dorsal 1 cm of the bony nasal septum and the rostrum were resected. A wide bilateral sphenoidotomy was completed and the sphenoid septa removed. Only the sellar floor’s mucosa and bony floor were removed. The dura was incised and the tumor exposed. Then, 30° and 45° endoscopes helped to complete the tumor resection.

### Endoscope-assisted microscopic mononostril transnasal paraseptal transsphenoidal approach

A speculum was inserted through one nostril. The turbinates were lateralized. The nasal septum was displaced to the contralateral side. The rostrum was drilled. The other steps were identical to E. Finally, angled endoscopes were introduced to find tumor remnants.

### Statistical analysis

The statistical analysis was performed with the R-programming language (version 4.0.3) (22). Fisher exact tests and chi-square tests were used for categorical data; the continuous variables were compared using *t*-tests and Wilcoxon tests. Shapiro–Wilk test was used to control the normal distribution of the continuous variables. The significance level was *p <*0.05.

## Results

### Patient-specific and surgical parameters

A total of 18 (E) and 15 (M) patients with a histopathological diagnosis of pituitary adenoma were eligible—four patients had a Rathke cleft cyst and were excluded, and three further cases were also ruled out from the analysis (due to wish or independent death). **Table 2** shows a comparison of the relevant patient-specific and surgical data of both groups. The M-surgeries’ duration was significantly shorter; further parameters were similar between the groups.

**Table 2:** Patient-specific and surgical data.

Aspect		E (*N* = 18)	M (*N* = 15)	*p*-value
Preoperative				
Age (years)		Mean: 53.5 (range: 25.9–80.9)	Mean: 57.6 (range: 34.3–82.6)	0.4475
Sex		8 female, 9 male, 1 transsexual	4 female, 11 male	0.4688^a^
BMI prior to surgery (kg/m^2^)		Mean: 29.7 (range: 21.6–41.4)	Mean: 26.8 (range: 20.4–34.7)	0.1226
Symptom leading to diagnosis	No symptoms (incidental)	16.7% (*N* = 3)	6.7% (*N* = 1)	0.6074
	Headache	44.4% (*N* = 8)	26.7% (*N* = 4)	0.4688
	Subjective visual complaint	72.2% (*N* = 13)	53.3% (*N* = 8)	0.3005
	Subjective endocrinological complaint	83.3% (*N* = 15)	60.0% (*N* = 9)	0.2395
	Cranial nerve palsy	5.6% (*N* = 1)	6.7% (*N* = 1)	1.0000
	Hydrocephalus	None	None	
	Apoplex	None	None	
Relevant preoperative comorbidity		77.8% (*N* = 14)	60.0% (*N* = 9)	0.4483
Intraoperative				
Surgical approach laterality		Bilateral	Unilateral	
Duration of surgery (min)		mean: 157 (range: 89–262)	mean: 118 (range: 85–162)	**0.003883**
Use of navigation		5.6% (*N* = 1)	None	
CSF-leak		22.2% (*N* = 4)	46.7% (*N* = 7)	0.1631
Lumbal drainage placement (intraoperative)		16.7% (*N* = 3)	13.3% (*N* = 2)	1.0000
Skull base reconstruction	Autologous fat tissue	38.9% (*N* = 7)	53.3% (*N* = 8)	0.4939
	Fibrin sealant patch^b^	22.2% (*N* = 4)	13.3% (*N* = 2)	0.6648
	Fibrin glue^c^	22.2% (*N* = 4)	46.7% (*N* = 7)	0.1631
	Gelatin foam^d^	61.1% (*N* = 11)	46.7% (*N* = 7)	0.4939
	Gelatin-thrombin matrix^e^	16.7% (*N* = 3)	13.3% (*N* = 2)	1.0000
	Nasoseptal flap	None	None	
Postoperative				
Hospital stay (days)		Mean: 7.7 (range: 4–24)	Mean 7.8 (range: 5–23)	0.9535
Follow-up period (years)		Mean: 7.0 (range: 1.2–13.9)	Mean: 5.5 (range: 1.0–14.0)	0.3513
Complications^e^	Total	16.7% (*N* = 3)	20.0% (*N* = 3)	1.0000
	CSF leak	5.6% (*N* = 1)	13.3% (*N* = 2)	0.5794
	Revision surgery	5.6% (*N* = 1)	6.7% (*N* = 1)	1.0000

a In this analysis, the transsexual individual’s gender was classified based on the gender identity at the time of surgery.

b TachoSil, Takeda GmbH, Konstanz, Germany.

c Tisseel, Baxter Deutschland GmbH, Unterschleißheim, Germany.

d Gelaspon, Bausch+Lomb GmbH, Berlin, Germany.

e FloSeal, Baxter Deutschland GmbH, Unterschleißheim, Germany.

f Endocrinological, ophthalmological, and rhinological issues are discussed separately. Statistically significant data was marked bold.

### Histology and morphology

The histological features were comparable (**Table 3**). The lesions’ presurgical volume was similar. After dichotomization into infiltrating versus non-infiltrating tumors, there were no significant differences.

**Table 3:** Parameters of lesions.

Morphology		E (N=18)	M (N=15)	p-value
**Tumor volume** (mm^3^)		mean: 8163.6 (range: 52.4-24683.4)	mean: 4536.88 (range: 500.9-10117.5)	0.5867

Diagnosis	Subtype	E (N=18)	M (N=15)	p-value
**Adenoma**	**non-functioning**	61.1% (N=11)	66.7% (N=10)	0.8368
	**ACTH-producing**	11.1% (N=2)	20.0% (N=3)	
	**PRL-producing**	11.1% (N=2)	6.7% (N=1)	
	**GH-producing**	16.7% (N=3)	6.7% (N=1)	
	**TSH-producing**	None	None	

Category	Grade	E (N=17)^†^	M (N=13)^†^	p-value
Cavernous sinus infiltration (Knosp)*	**0**	17.6% (N=3)	38.5% (N=5)	0.2942
	**1**	5.9% (N=1)	15.4% (N=2)	
	**2**	5.9% (N=1)	None	
	**3A**	47.1% (N=8)	15.4% (N=2)	
	**4**	23.5% (N=4)	30.8% (N=4)	
**Sellar floor infiltration (Hardy)**	**0**	17.6% (N=3)	15.4% (N=2)	0.9212
	**I**	None	7.7% (N=1)	
	**II**	11.8% (N=2)	15.4% (N=2)	
	**III**	47.1% (N=8)	46.2% (N=6)	
	**IV**	23.5% (N=4)	15.4% (N=2)	
**Suprasellar tumor expansion (Hardy)**	**0**	23.5% (N=4)	23.1% (N=3)	0.05196
	**A**	29.4% (N=5)	46.2% (N=6)	
	**B**	5.9% (N=1)	30.8% (N=4)	
	**C**	35.3% (N=6)	None	
	**D**	5.9% (N=1)	None	

*If the Knosp grade was different between both sides, the lesion was classified according to the higher grade.

†In 3 cases the preoperative classification of tumor infiltration was not clearly possible.

### Complications

Complications occurred in three E and three M patients (total: 18.2%, intercohort rate: *p* = 1.0000, OR = 0.80). The rhinological/endocrinological aspects are discussed separately. Postoperative CSF leak emerged in 9.1% (*N* = 3). The morbidities had no long-term medical consequences. There was no related mortality.

A total of 15 individuals (83.3%) of E were without complications: one patient underwent surgical revision due to CSF leak (5.6%), and another’s epistaxis was treated conservatively (5.6%). Another patient needed prolonged intensive care due to Addisonian crisis and pneumothorax (5.6%).

A total of 12 patients (80.0%) of M were without complications. A postoperative CSF leak (6.7%) was treated with lumbal drainage. Another patient underwent early revision surgery (6.7%) due to residual tumor and later again due to paraumbilical hemorrhage (6.7%) and CSF leak. One individual complained of nostril skin tear (6.7%).

### Extent of resection

Angled optics revealed tumor in 63.6% (E; *N* = 7 from 11) and 58.3% (M; *N* = 7 from 12) where GTR was set as a goal of the surgery. The endoscopic or microsurgical technique carried no significant long-term recurrence rate difference in these subgroups (*p* = 1.0000, OR = 1.20). Independently from any goal, pure surgical long-term tumor-free state was achieved in E in 61.1% (*N* = 11), stable residuum in 16.7% (*N* = 3), and recurrence in 22.2% (*N* = 4, here adjuvant therapy was started). In M, pure surgical long-term tumor-free state was described in 86.7% (*N* = 13), stable residuum in 6.7% (*N* = 1), and recurrence in 6.7% (*N* = 1, here revision surgery was later performed). All patients underwent solo surgery with one M exception. The main results are detailed in **Table 4**.

**Table 4:** Outcome data.

Feature		E (*N* = 18)	M (*N* = 15)	Comments and/or *p*-value
Extent of resection				
All patients	Long-term tumor-free state (without adjuvant therapy or revision surgery)	61.1% (*N* = 11)	86.7% (*N* = 13)	0.1336
	Long-term tumor-free state (with adjuvant therapy or revision surgery)	72.2% (*N* = 13)	93.3% (*N* = 14)	0.1861
	Long-term stable residual tumor	16.7% (*N* = 3)	6.7% (*N* = 1)	0.6074
	Recurrence (anytime)	22.2% (*N* = 4)	6.7% (*N* = 1)	0.3457
	Revision surgery	None	6.7% (*N* = 1)	0.4545
	Chemotherapy	5.6% (*N* = 1)	None	1
	Irradiation	16.7% (*N* = 3)	None	0.233
	Final tumor volume (mm^3^)	Mean: 87.3 (range: 0–686.1)	Mean: 34.2 (range: 0–478.5)	0.1093
Patients with GTR as initial goal	Total	61.1% (*N* = 11)	80.0% (*N* = 12)	0.2828
	Intraoperative (angled) endoscopy reveals residual tumor	63.6% (*N* = 7)	58.3% (*N* = 7)	0.6534
	Intraoperative (angled) endoscopy shows no residual tumor	36.3% (*N* = 4)	41.7% (*N* = 5)	
	Long-term tumor free-state	81.8% (*N* = 9)	83.3% (*N* = 10)	*p* = 1.0000, OR = 1.2
Endocrinological aspects				
Anterior pituitary function: prior surgery	Dysfunctional	94.4% (*N* = 17)	86.7% (*N* = 13)	However, only 52.5% (*N* = 21) were non-productive adenomas
	Normal	5.6% (*N* = 1)	13.3% (*N* = 2)	
Anterior pituitary function: final exam	Improved	61.1% (*N* = 11)	46.7% (*N* = 7)	Mostly subclinical deficits. No significant intercohort difference at the latest exam. Final anterior pituitary function: 1.0000
	Unchanged	27.8% (*N* = 5)	26.7% (*N* = 4	
	Deteriorated	11.1% (*N* = 2)	26.7% (*N* = 4)	
Anterior pituitary function: final exam	Dysfunctional	77.8% (*N* = 14)	80.0% (*N* = 12)	
	Normal	22.2% (*N* = 4)	20.0% (*N* = 3)	
Subgroup of patients with hormone excess	Complete healing	66.7% (*N* = 4)	66.7% (*N* = 2)	Similar rate of patients without substitution therapy at the latest exam: 1.0000. From 7 hormone-producing E cases, 6 had manifested disease. From 4 hormone-producing M cases, 3 had manifested disease
	Incomplete healing	33.3% (*N* = 2)	33.3% (*N* = 1)	
	No healing	None	None	
Diabetes insipidus	Temporary	11.1% (*N* = 2)	None	0.4886
	Permanent	None	None	
SIADH	Temporary	None	6.7% (*N* = 1)	0.4545
	Permanent	None	None	
Rhinological aspects				
Subjective symptoms	Present prior surgery	38.9% (*N* = 7)	20.0% (*N* = 3)	
	Present at the latest exam	11.1% (*N* = 2)	13.3% (*N* = 2)	
Sense of smell (objective test), final exam	Normosmia	38.9% (*N* = 7)	66.7% (*N* = 10)	No significant intercohort difference of the latest objective (0.2365) and subjective (0.6483) tests. No significant difference between one’s latest subjective and objective olfaction (0.3326)
	Hyposmia	38.9% (*N* = 7)	20.0% (*N* = 3)	
Sense of smell (subjective), final exam	Normosmia	55.6% (*N* = 10)	73.3% (*N* = 11)	
	Hyposmia	22.2% (*N* = 4)	13.3% (*N* = 2)	
Rhinomanometry	Latest exam			0.8774
Ophthalmological aspects				
Reduced visual acuity	Prior surgery	38.9% (*N* = 7)	60.0% (*N* = 9)	0.4967
	Postoperative	None	13.3% (*N* = 2)	
Tumor-related visual field defect	Prior surgery	55.6% (*N* = 10)	40.0% (*N* = 6)	1.0000
	Improved (incomplete)	20.0% (*N* = 2)	16.7% (*N* = 1)	
	Normalized	70.0% (*N* = 7)	66.7% (*N* = 4)	
	Persisted	10.0% (*N* = 1)	16.7% (*N* = 1)	

### Endocrinological outcome

There were 94.4% (E; *N* = 17) and 86.7% (M; *N* = 13) patients who had preoperative anterior pituitary dysfunction (**Table 4**). However, 63.6% (*N* = 21) were non-functioning adenomas. Finally, four (E; 22.2%) and three (M; 20.0%) patients had normal anterior pituitary function. The deteriorations were mostly subclinical without hormonal substitution.

**Figure 1:**
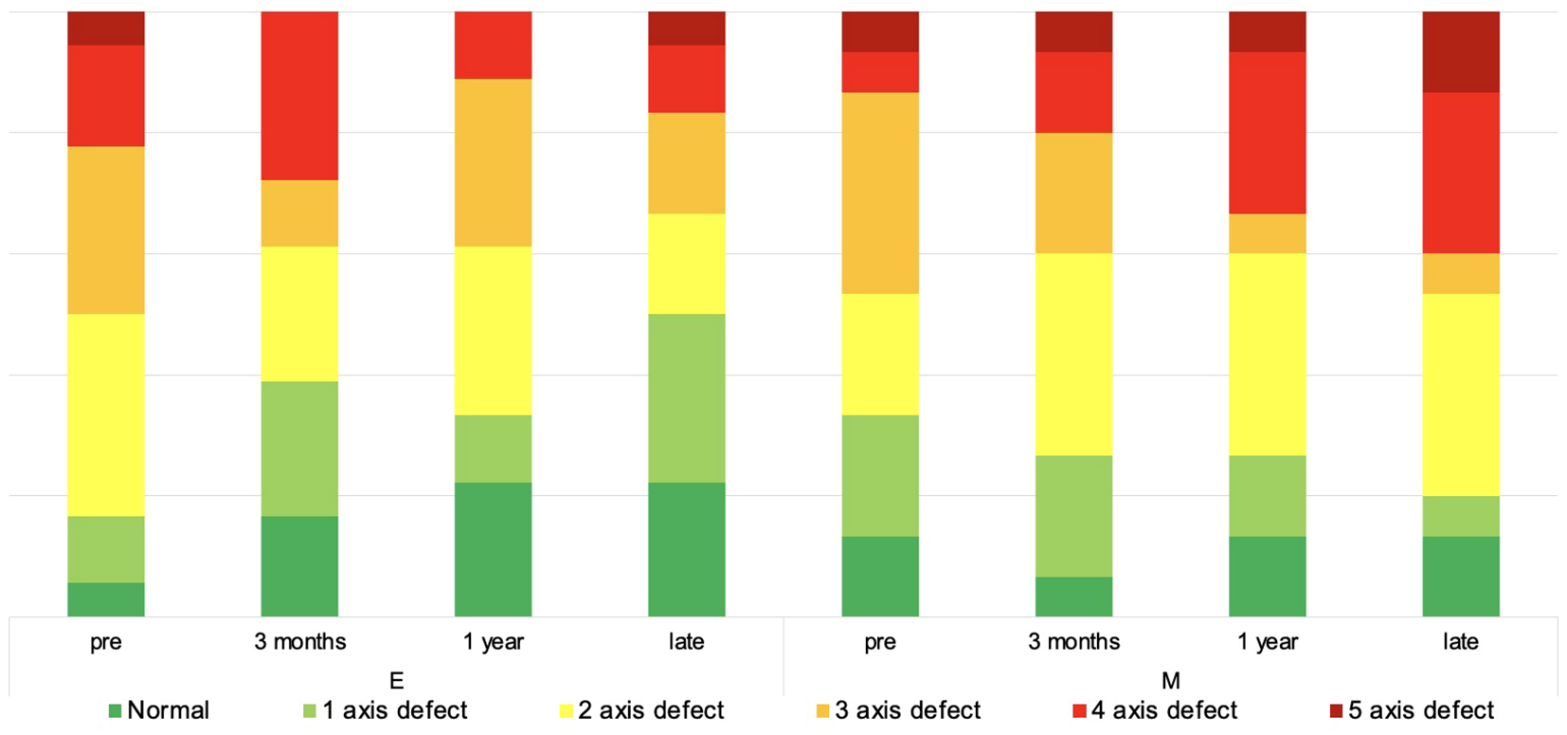
Anterior pituitary lobe function over time. The diagram depicts the non-continuous development of anterior pituitary lobe function over time.

**Table 5** demonstrates anterior lobe function dynamics. Furthermore, 83.3% (*N* = 5 from 6) with deterioration already presurgically presented multiaxial deficiency. Endoscopic technique was related to a higher probability of anterior pituitary lobe function improvement (OR = 1.60). **Figure 1** demonstrates the pituitary function over time. There was neither a significant intercohort difference at the latest follow-up (*p* = 0.3166) nor in the overall trends (*p* = 0.2583).

**Table 5:** Anterior pituitary lobe function.

Trend score^a^	E (*N* = 18)	M (*N* = 15)	Comment
+3	11.1% (*N* = 2)	None	No significant intercohort difference of anterior pituitary function trends, *p* = 0.2583. Endoscopic technique was related to a higher probability of anterior pituitary improvement (OR = 1.60)
+2	16.7% (*N* = 3)	None	
+1	33.3% (*N* = 6)	46.7% (*N* = 7)	
0	27.8% (*N* = 5)	26.7% (*N* = 4)	
-1	11.1% (*N* = 2)	6.7% (*N* = 1)	
-2	None	20.0% (*N* = 3)	

a Difference of the anterior pituitary axes at the latest and the preoperative time point. Positive score, improvement; zero, unchanged; negative, deterioration—for example, an initial 4-axis-hypopituitarism (-4) with a final 2-axis-hypopituitarysm (-2) results in a score of +2.

### Rhinological outcome

We recognized no significant intercohort differences of rhinological aspects. There were no relevant postsurgical pathologies (**Table 4**).

### Ophthalmological outcome

The improvement of the visual acuity and visual field, respectively, were similar in both groups (**Table 4**). There was no deterioration of any visual function.

### Quality of life

**Figure 2:**
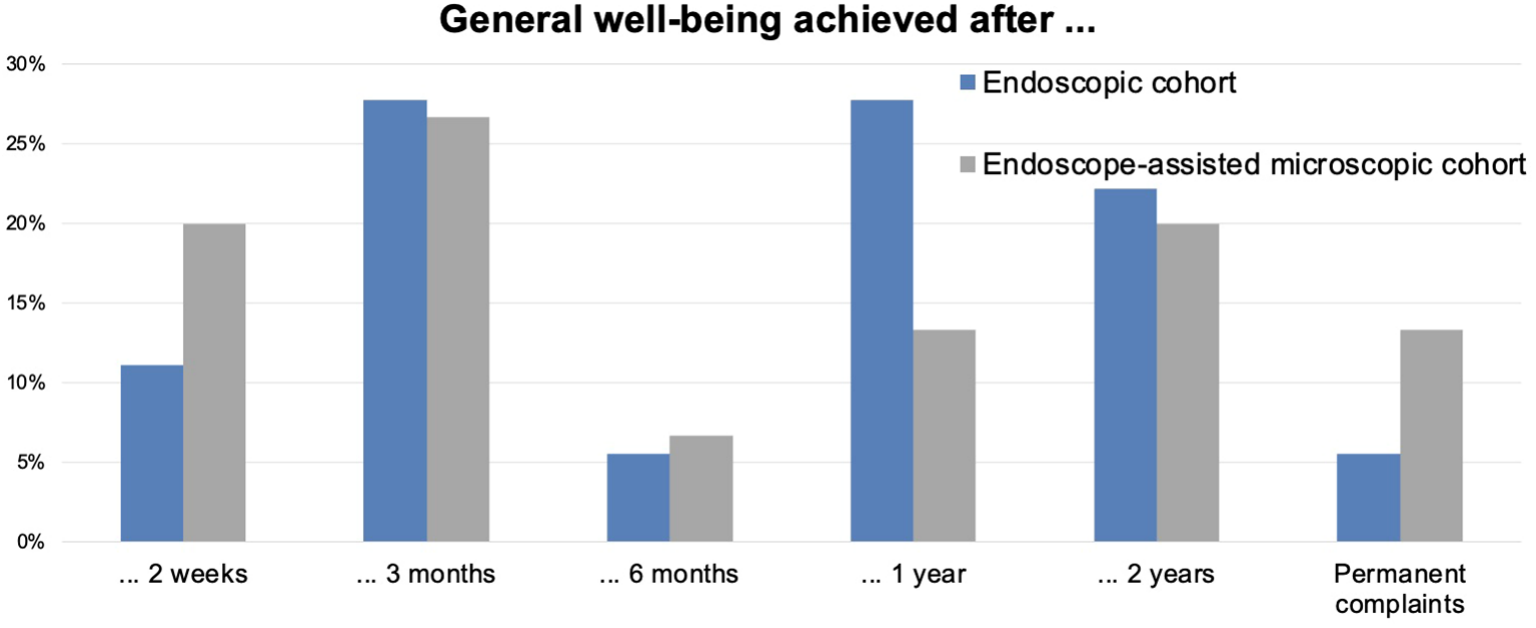
Achievement of overall well-being over time. The diagram depicts the non-continuous time point when symptom-free well-being was achieved. The values are in %.

**Figure 3:**
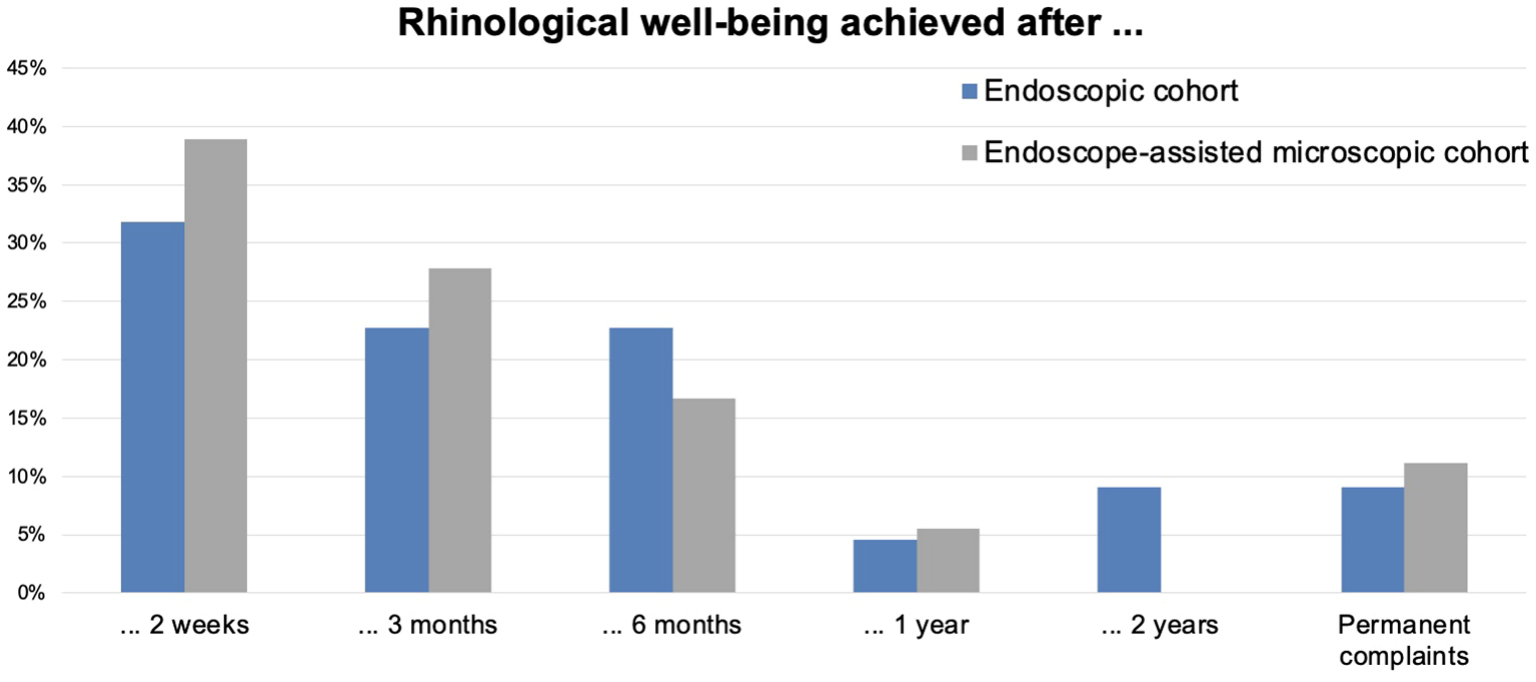
Achievement of rhinological well-being over time. The diagram depicts the non-continuous time point when symptom-free well-being was achieved. The values are in %.

All patients had presurgical complaints (**Supplementary Table S1**). Finally, 94.4% (E; *N* = 17) and 86.7% (M; *N* = 13) achieved subjective well-being (**Figure 2**). Contrary to the early rhinological reconvalescence (**Figure 3**), the general recovery showed unspecific distribution. The working status data was comparable in both groups.

**Figure 4:**
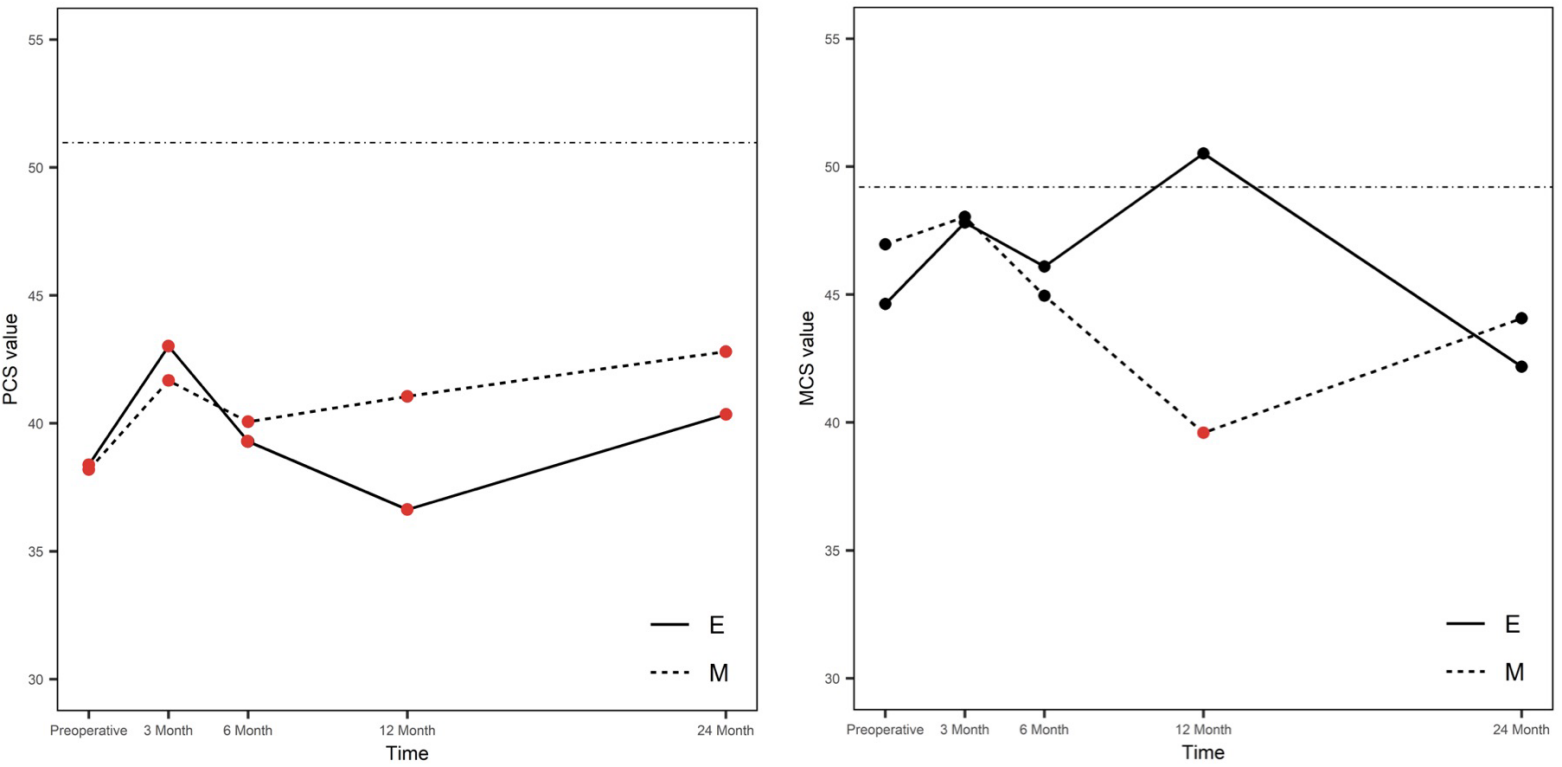
Mental and physical scores of SF-36 over time. E, endoscopic group; M, microsurgical group; MCS, mental component summary score; PCS, physical component summary score; continuous line, normal value of German population. Higher scores indicate a higher quality of life. Significant (red dots) and non-significant (black dots) results compared to the normal value are distinguished. We compared our data to the healthy German population (46) (straight line).

The data of SF-36 was available in 15 E and 13 M cases. The SF-36 summarizes eight domains in the physical (PCS) and mental (MCS) score. The MCS showed no relevant changes across time between the groups and compared to the normal value (**Figure 4**). The PCS was significantly worse than the normal value but without difference between the groups across time.

SNOT-22 results were available in seven E and eight M cases. The average trend of the preoperative and final scores was similar, *p* = 0.5474. Moreover, 14 E and 11 M patients filled the questionnaire 1 year after surgery; there was no relevant difference (*p* = 0.8588).

### The value of microsurgical pituitary surgery

Endoscopy revealed residual tumor in 46.7% of cases (M group; *N* = 7) that had not been detected using the microscope alone. These remnants were subsequently removed with the endoscope, and follow-up MRI confirmed no recurrence in these patients.

We hypothesized that endoscopy was not added in M. Tumor-free state without adjuvant therapy would be then long-term maintained in favor of the endoscopic group (*N* = 11; 61.1%) compared to the pure microscopic cohort (*N* = 6, 40.0%; *p* = 0.4939, OR = 1.76). As a comparison, the factual OR in this aspect was 0.19 between the endoscopic and endoscope-assisted microsurgical groups. Endoscopy seemed also advantageous concerning the achievement of the initial surgical goal (*p* = 0.08272, OR = 3.80) and the recurrence rate without (*p* = 0.08272, OR = 0.26) as well as with adjuvant therapy (*p* = 0.1264, OR = 0.24). Consequently, added endoscopy relevantly improved the surgical outcome.

In eight M cases (53.3%), endoscopy revealed no residual tumor. Here a pure microsurgical manner *would have been* sufficient. We compared this subgroup (M*) to the E cohort: there was no significant difference of the trends in the anterior pituitary lobe function (*p* = 0.2810), the trends of the objective (*p* = 0.2433), and subjective olfaction (*p* = 0.5132) or the ophthalmologic outcome (*p* = 0.6655). At 24 months after surgery, the PCS and MCS of SF-36 did not differ significantly between the E and M* groups (*p* = 0.4561 and 0.4598, respectively). **Table 6** presents a summary of our most relevant findings along with their statistical power.

**Table 6:** Key observations.

Aspect	Advantageous group	Statistical rationale	Endoscopic group was compared with
Surgical duration	Endoscope-assisted microsurgical	*p* = 0.003883	Endoscope-assisted microsurgical
Long-term tumor recurrence	Endoscopic	OR = 0.24	Microsurgical^a^
Long-term tumor-free state	Endoscopic	OR = 1.76	Microsurgical^a^
Long-term achievement of any initial surgical goal	Endoscopic	OR = 3.80, *p* = 0.08272	Microsurgical^a^
Long-term improvement of anterior pituitary lobe function	Endoscopic	OR = 1.60	Endoscope-assisted microsurgical

a Calculated as endoscopic inspection would not have been performed during surgery.

## Discussion

The superiority of endoscopy over microsurgery is a recurring statement (9, 10, 12, 20, 23, 24). This conclusion is primarily drawn from retrospective case series and meta-analyses that lack randomization and offer less control of confounding variables. Consequently, controversies regarding surgical outcomes persist, underscoring the need for randomized studies (21, 25).

There are only a few prospective (26–29) or randomized (27, 30) comparative studies. We believe that our study design offers valuable contributions by minimizing potential biases and presenting long-term data that complement the existing evidence. Additionally, the brief patient enrollment period reduces the influence of external factors such as changes in technology and medical care over the course of our series. Our review underlined the need for prospective (to reduce observer bias), randomized (no surgeon’s influence on division), and long-term (late morbidity/recurrence recognition) comparative studies which implement subjective PROMs.

### Surgical parameters and complications

Razak et al. (31) reported increased endoscopic surgical duration, contrary to others (16, 27, 30). Due to the time-consuming nasal endoscopic phase, our M procedures were significantly shorter. At the time of our study, surgery starting point was defined by the beginning of anesthesia. This explains the relatively long durations. Contrary to us, others found shorter hospital stay in E cases (11, 12, 16, 30, 32).

We observed few complications without intercohort difference. The complication rates are around 20% (17, 32). Li et al. (33) showed comparable results of epistaxis, CSF leak, and meningitis. Castanão-Leon et al. (12) explain the lower endoscopic incidence of CSF-leak due to better visualization; however, others described higher rates (8, 34). Major complications are rare (6, 14, 15, 35).

### Extent of resection

Endoscopy is believed to reduce recurrence (8, 11, 12, 36, 37). This advantage was significant in the study of Castanão-Leon et al. (12) (63.9% versus 42.2%). Recent metaanalyses found similar GTR rates of both techniques (21, 25). Our final rate of tumor-free state was 72.2% (endoscopy), 93.3% (endoscope-assisted microscopy), and 40.0% (hypothetical pure microscopic group). Recurrence usually appeared late, often after many years.

### Endocrinological outcome

Anterior pituitary dysfunction occurs in 3%–6% (microsurgery) and 1%–3% (endoscopy) (16, 38, 39). Eseonu et al. (16) found comparable risks of hypopituitarism. According to Asemota et al. (35), the endoscopic rates of DI and SIADH were significantly higher. D’Haens et al. (34) described similar hypersecretion remission rates of 50% (microscopy) and 63% (endoscopy).

Due to the superior magnification with the endoscope, the normal pituitary can be protected, making hypopituitarism less likely (28, 30). We observed anterior lobe deterioration in 11.1% (E) and 26.7% (M). All of these patients already showed preoperative multiaxial deficiency. What could explain the differences? Firstly, our testing often revealed subclinical presurgical hormonal deficits which are not commonly registered. Secondly, the remission of pituitary function can be delayed.

In our series, the intercohort trends of anterior pituitary function were comparable. Contrarily, Castanão-Leon et al. (12) described better endoscopic remission rates (except Cushing’s disease). ACTH-producing microadenomas reportedly have outstanding microsurgical results; however, Bora et al. (40) found better endoscopic remission rates. Razak et al. (31) reported higher remission rates of functional adenomas after endoscopic surgery (94% vs. 57%). Phan et al. (41) described similar rates of endocrine remission in acromegaly patients.

### Visual outcome

Endoscopy is thought to be advantageous in this field (12). In accordance with others (14, 25, 30), we found no significant differences. Asemota et al. (35) described visual field defects (4.7%), paralytic strabismus (2.2%), and diplopia (1.6%) without significant intercohort difference. Another study (11) postulates that endoscopy may provide better visual outcomes with an improvement rate of 71% vs. 56%. Bryl et al. (42) described a benefit for the endoscopic approach concerning visual QOL.

### Rhinological outcome

Four of our patients (12.1%) had late rhinological complaints; three of them would otherwise qualify as “surgical success” without tumor recurrence. PROMs should be implemented in the daily routine as they revealed preoperative sinonasal complaints in a quarter of our patients.

Endoscopy is considered to be less traumatic to the olfactory function as no retractor is used (26, 43). Nevertheless, Bryl et al. (42) found similar rhinological QOL data. Osborne et al. (44) found comparable long-term outcomes.

### Quality of life

Kuan et al. (45) found no significant endoscopic surgery-related changes of SF-36 scores. Pledger et al. (15) identified no relevant differences of SF-36 scores between endoscopic and microscopic cohorts. SF-36 is a useful but general tool which should be complemented by specific pituitary-relevant aspects. We believe that our results are not specific as MCS and PCS relevantly differ. Another questionnaire (**Supplementary Table S1**) revealed long-term patients’ satisfaction in 86.7% M and 94.4% E.

### Statistical power and limitations

The higher number of male subjects in the M group is a possible limitation; however, the gender distribution differences were not statistically significant. Similarly, the differences of the Knosp and Hardy grades were statistically not significant, but they may have had a subtle influence on the results.

The main limitation of this single-center, single-surgeon analysis is the low case number. However, we believe that our study design complements the existing literature and adds value by applying PROMs, minimizing biases and contributing long-term data (up to 14 years). We included consecutive cases without patient selection bias. The outcome assessment was blinded; the patients were not aware of their group allocation. Most prospective trials report of shorter follow-up periods (e.g., 6 months) (25). We recognized delayed surgical consequences emphasizing the necessity of long-term observations. A larger sample size would allow more profound conclusions. Meta-analyses provide data of high numbers of patients; however, different study methods and interpretations reduce their feasibility. Therefore, future large-scale, randomized analyses are necessary to confirm our results.

## Conclusions

Both endoscopic and endoscope-assisted microsurgical techniques provide good long-term endocrinological, rhinological, and ophthalmological results. Endoscopy improved the rate of long-term achievement of the initial surgical goal, the tumor-free state, and the anterior pituitary lobe function.

## Data Availability

The raw data supporting the conclusions of this article will be made available by the authors, without undue reservation.
