# Supplementary material for: Comparison of endoscopic and endoscope-assisted microscopic transsphenoidal surgery for pituitary adenoma resection: a prospective randomized study
Source: Front Endocrinol (Lausanne). 2025 Jul 22;16:1552526. doi: 10.3389/fendo.2025.1552526 (PMC12322937; doi:10.3389/fendo.2025.1552526)
Supplement: Supplementary file 1 [file Table1.docx]

| **Supplemental Table 1. Standardized questionnaire** | |
| --- | --- |
| **Group** | **Items** |
| **General** | subjective complaints, concentration issues, sleep habits, work performance, Karnofsky-score, list of medication |
| **Neurological** | awakeness, headache, dizziness, emesis, gait difficulty, paresis, any deficits in the detailed neurological physical examination |
| **Ophthalmological** | visual loss, pupillomotor and oculomotor disorders, visual field deficits, diplopia |
| **Endocrinological** | reduced performance, fatigue, lactation, menstruation issues, weight changes, sexual problems |
